# Atorvastatin and flaxseed dietary treatments improve dyslipidemia and liver injuries in a diet-induced rat model of non-alcoholic fatty liver disease

**DOI:** 10.22038/ajp.2024.25220

**Published:** 2025

**Authors:** Zahra Eslami, Hamidreza Joshaghani, Abdorreza Eghbal Moghanlou, Alireza Norouzi, Seyed Javad Mirghani

**Affiliations:** 1 *Department of Clinical Biochemistry, Hamadan University of medical science, Hamadan, Iran*; 2 *Laboratory sciences research center, Golestan University of Medical Sciences, Gorgan, Iran*; 3 *Istanbul esenyurt University, Physical Education and Sports High School, Coaching Education Department, Istanbul, Turkey*; 4 *Golestan Research Center of Gastroenterology and Hepatology, Golestan University of Medical Sciences, Gorgan, Iran*; 5 *Shahid Mirghani Research Institute, Gorgan, Golestan, Iran*

**Keywords:** Statin, Flax, Liver, Linseed Oil, Steatosis

## Abstract

**Objective:**

Non-alcoholic fatty liver disease (NAFLD) as the most common chronic liver disease is associated with metabolic disorders including dysregulated lipid and glucose metabolism. There is no approved drug treatment for NAFLD; thus, new therapies are needed. We studied the antidyslipidemic effects of atorvastatin and/or possibly hepatoprotective effects of flaxseed/ flaxseed oil in a rat model of NAFLD.

**Materials and Methods:**

Fifty-six male Wistar rats were divided randomly into seven groups: 1) control, 2) high-fructose diet (HFD), 3) HFD +atorvastatin (20 mg/kg), 4) HFD+ flaxseed (40 g/kg), 5) HFD+ flaxseed oil (40 mg/kg), 6) HFD+flaxseed (40 g/kg) + atorvastatin (20 mg/kg) and 7) HFD+flaxseed oil (40 g/kg) +atorvastatin (20 mg/kg). The interventions were done for 23 weeks, after which anthropometric indices, lipid profile, liver enzymes, fasting blood glucose, and kidney indices were analyzed. Scoring of hematoxylin-eosin-stained liver sections was used to assess the severity of NAFLD.

**Results:**

All the treatments reduced mesenteric fat mass, and the amount of fat around the liver, except in HFD+ flaxseed +atorvastatin group. The interventions improved NAFLD activity score, which considers steatosis, lobular inflammation, and hepatocyte ballooning. However, atorvastatin was most efficient in reducing inflammation and hepatocyte ballooning. While atorvastatin reduced only Gamma-glutamyltransferase (GGT) levels, flaxseed or flaxseed oil mono- and combination therapies reduced serum levels of all liver enzymes. The interventions improved the serum lipid profile and all, except atorvastatin decreased fasting blood glucose.

**Conclusion:**

Flaxseed therapies improved NAFLD-associated liver injuries and dyslipidemia, while atorvastatin mostly reduced hepatocyte ballooning and lobular inflammation.

## Introduction

Non-alcoholic fatty liver disease (NAFLD) is considered the hepatic manifestation of metabolic syndrome (Ford 2005; Yki-Järvinen 2014). NAFLD is the most common cause of liver dysfunction globally, which affects ~25% of the adult population and up to 90% of the obese population (Huang et al. 2021). NAFLD is defined by significant lipid accumulation in the hepatocytes (>5% fat) when the patients do not consume alcohol excessively (Benedict and Zhang 2017). 

The pathological conditions of NAFLD range from simple steatosis to nonalcoholic steatohepatitis (NASH), further developing into cirrhosis, and hepatocellular carcinoma (HCC). NASH is an inflammatory form of NAFLD, where inflammation causes liver damage and scarring (Beheshti Namdar et al. 2023; Chen and Yeh 2021). Fibrosis can progress over time, resulting in severe scarring of the liver and likely leading to the development of cirrhosis. Individuals with cirrhosis have a higher risk of developing hepatocellular carcinoma (HCC) and severe liver failure (Farrell et al. 2008). 

The treatment options for NAFLD are currently focused on diet- and exercise-induced weight loss. However, the effectiveness of lifestyle changes is frequently compromised by the low adherence of the patients to the treatment (Romero-Gómez et al. 2017). To date, there are no approved drugs to treat NAFLD, which justifies the search for new and safe therapeutic alternatives that could minimize the damage caused by NAFLD. Given that around 70% of patients with NAFLD also have associated dyslipidemia (Hyogo et al. 2008), therapy with an anti-dyslipidemic drug could be a viable treatment option in the long term.

Atorvastatin is an enzyme of the hepatic cholesterol biosynthesis pathway, and the most widely prescribed statin medication in the world, and it is used to treat hypercholesterolemia and mixed dyslipidemia (Rasekh et al. 2022; Ye et al. 2015). Besides its cholesterol-lowering effects, atorvastatin has already been demonstrated to have anti-inflammatory, anti-oxidative, and anti-fibrogenic effects in clinical and pre-clinical studies (Gao et al. 2017; Lastuvkova et al. 2021). These physiological properties can play an important role in preventing the onset of NAFLD. Yet, some studies have shown that statins can cause mild liver injuries leading mostly to an increase in serum levels of liver enzymes (Averbukh et al. 2022).

It has been reported that flaxseed (*Linum usitatissimum*), a vegetal source of omega-3 (n–3) fatty acid -linolenic acid (ALA, C18:3n–3), causes significant health benefits in many chronic diseases, like cancer (DeLuca et al. 2018), cardiovascular diseases (Parikh and Pierce 2019), and diabetes (Prasad and Dhar 2016). Flaxseed and flaxseed oil improve lipid homeostasis by lowering cholesterol levels (Parikh and Pierce 2019; Yang et al. 2009), as well as by having antioxidant (Prasad 2000) and anti-inflammatory (Dupasquier et al. 2007) effects in metabolic diseases. Besides, flaxseed has demonstrated prominent hepatoprotective properties, thus possibly playing a key protective role in NAFLD (Parker et al. 2012). ALA seems to be better absorbed when ingested as flaxseed oil compared to whole or ground flaxseeds (Austria et al. 2008). 

As statins are well-established medications to reduce cholesterol, lipoprotein-associated, and triglyceride (TG) levels and improve hepatic health, and flaxseed is shown to exert possible hepatoprotective effects, a therapeutic strategy using their combination could be beneficial for NAFLD management. Therefore, we assessed the effects of atorvastatin and flaxseed (whole or oil) in a rat model of NAFLD and determined whether the combination treatment could improve NAFLD-related parameters better than the monotherapies. 

## Materials and Methods

### Experimental animals

Fifty-six male Wistar rats (250-300 g) were kept in standard cages under a 12:12 hr light/dark cycle at 20-24°C. The rats were first fed a chow diet and had access to *ad libitum* drinking water. They received humane care following the guidelines of Laboratory Animal Care, formulated by the National Society for Medical Research and the Guidelines for the Care and Use of Laboratory Animals by the Institute of Laboratory Animal Resources (NIH Publication No. 86–23, revised in 1985).

### Experimental design

The animals were let acclimatize for one week before starting the experiment. Animals were divided randomly into seven groups (eight rats per group): 1) control (normal diet, ND), did not undergo induction of NAFLD and received chow diet, 2) high-fructose diet (HFD) control, received 55% fructose solution and 0.1 ml/kg CCl_4_ (1:4 V/V dissolved in olive oil), 3) HFD+ Atorvastatin 20 mg/kg (diluted in 6% dimethyl sulfoxide (DMSO)) (HFD+ ATO 20), 4) HFD+ flaxseed 40 g/kg (HFD+F 40), 5) HFD+ flaxseed oil 40 mg/kg (HFD+ FO 40) (EL-Sayeda 2014), 6) HFD+ flaxseed 40 g/kg+ Atorvastatin 20 mg/kg (HFD+ F 40+ ATO 20), and 7) HFD+ flaxseed oil 40 mg/kg + Atorvastatin 20 mg/kg (HFD+ FO 40+ ATO 20). Flaxseed and flaxseed oil were purchased from Abkar Golestan Agroindustry Company (VERJEN), Golestan, Iran. The diet interventions lasted for 23 weeks.

### Induction of NAFLD

NAFLD was induced in rats by administering fructose by gavage and injecting of 0.1 ml/kg CCl_4_ (1:4 V/V dissolved in olive oil), intraperitoneally for 15 weeks as described previously (Eslami et al. 2021; Eslami et al. 2023). Fructose (Zar Grain Refinery, Alborz, Iran) solution was prepared at 55% dissolved in water.

### Tissue preparation

Following 12 hr of fasting, anesthetization was done with ketamine 50 mg/kg and xylazine 5 mg/kg (Merck, Germany), intraperitoneally. Liver tissues were removed and hepatic sections were fixed in formaldehyde 4% and embedded in paraffin for light microscopy assessment.

### Histopathology

In the hematoxylin and eosin–stained (H&E) sections, the histological characteristics were classified into three categories: hepatocellular injury, steatosis, and inflammation. Each sample was analyzed by a professional histopathologist by the stereological method of grid-point counting to measure the liver fat content (STEPanizer stereology version 1) (Elias et al. 1971; Tschanz et al. 2011). Steatosis (%) grade was as follows 0: <5, 1: 5-33, 2: 34-66, and 3: > 66. Lobular inflammation was graded as 0: none, 1: in < 2 foci, 2: 2-4 foci, and 3: > 4 foci. Hepatocyte ballooning was scored as 0: none, 1: few ballooned cells, and 2: several ballooned cells. Further, NAFLD activity score (NAS) was used to clarify the liver disease stage in rats, offering an overall diagnosis for each case as NASH (score ≥5), borderline (score 3-4), or not NASH (score 0-2) (Kleiner et al. 2005). 

### Analyses of clinical biochemical parameters

Animals’ weight was monitored weekly. After the week 23, following 12 hr fasting and under anesthesia (ketamine 50 mg/kg and xylazine 5 mg/kg (Merck, Germany) intraperitoneally), blood samples (5 ml) were collected by cardiac puncture. The lipid profile included analyses of total cholesterol, triglycerides, and high-density lipoprotein cholesterol (HDL-Chol). Measurements of liver enzymes included aspartate aminotransferase (AST), alanine aminotransaminase (ALT), gamma-glutamyl transferase (GGT), and alkaline phosphatase (ALP). In addition, fasting blood glucose levels were measured. All were assayed using specific enzymatic kits following the manufacturer’s instructions (Pars Azmoon., IRAN) (Autoanalyzer BT 3500, Med system, USA).

### Ethical Approval

 This research was done based on the Guidelines for the Care and Use of Laboratory Animals (NIH Publication No. 85–23, revised in 1996). The research was approved by the local ethics committee (IR.GOUMS.REC.1397.274).

### Statistical analyses

The sample size was determined using G*Power software. We considered significance level (α) at 0.05 and power (1 - β) at % 80 to determine sample size. The statistical analyses were done with GraphPad Prism 9.1.0. The normal distribution of the data was assessed by the Shapiro-Wilk test. For group comparisons, we used One-Way ANOVA and Tukey post hoc test. The statistical significance was fixed at p<0.05

## Results

### The diet interventions efficiently reduced the amount of mesenteric fat and fat around the liver

**Figure 1:**
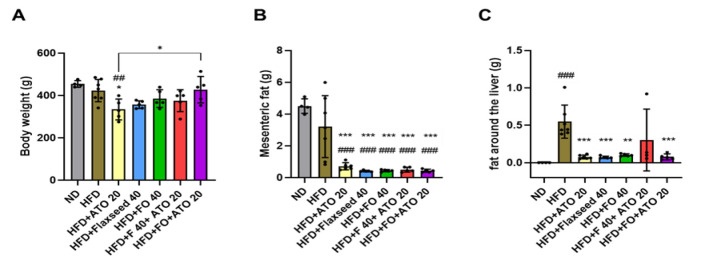
Body weight (A) as well as the mass of mesenteric fat (B) and fat around the liver (C) in the study groups. ND: Normal diet, HFD: High fat diet, ATO 20: atorvastatin 20 mg/kg, F: Flaxseed 40 g/kg, FO 40: Flaxseed oil 40 mg/kg. The results are expressed as mean ± SD for each group. Individual data points within the columns are indicated with dots. ###p<0.001 compared to ND, ***p<0.001 compared to HFD. The lines connecting the columns indicate that groups differ from each other, and * denotes for p<0.05.

 At the end of the intervention (23 weeks), anthropometric and biochemical measures were analyzed. The Atorvastatin monotherapy group weighed less than the HFD and ND groups (p<0.05 for both, Fig. 1A). The other HFD groups did not differ from ND group in body weight (Figure 1A). However, the HFD+FO 40+ATO 20 group weighed more than the HFD+ATO 20 (p=0.045). All intervention groups had a lower mesenteric fat mass than the ND and HFD groups (p<0.001 for all, Figure 1B). Although the weight of the liver did not differ between the study groups (data not shown), the amount of fat around the liver was higher in the HFD group than in the ND group (p=0.0003, Figure 1C). Importantly, all intervention groups had a lower amount of fat around the liver than the HFD group (p<0.001 for all, Figure 1C), except HFD+FO 40+ATO 20 group.

### The diet interventions improved NAFLD activity score, and atorvastatin was most efficient in reducing inflammation and hepatocyte ballooning

**Figure 2:**
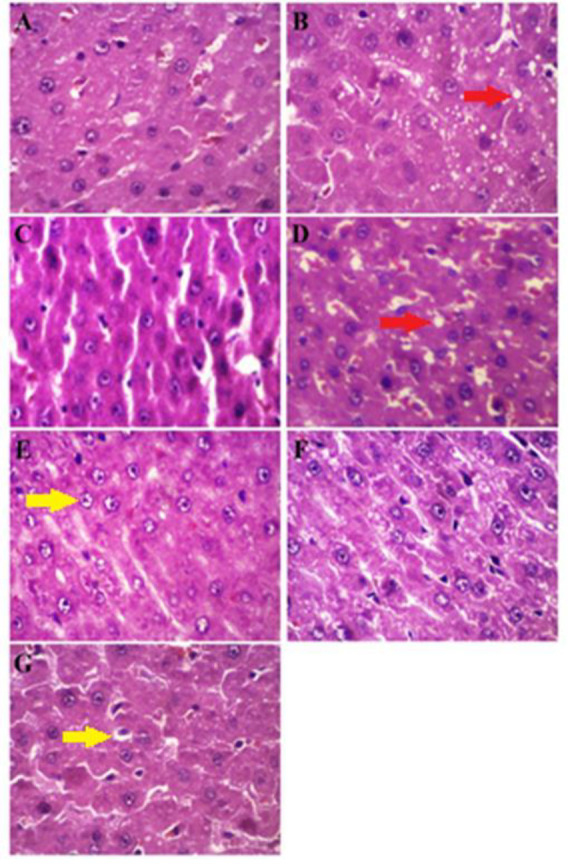
Representative of histological indices (Red: fat droplets, Yellow: inflammation and ballooning of the hepatocytes) of H&E staining images of the liver sections in the study groups (scale bar= 450 μm, × 400 magnification). A) CTL, B) HFD, C) HFD + ATO 20, D) HFD + F40, E) HFD + FO 40, F) HFD + F40 + ATO 20, and G) HFD + FO 40 + ATO 20. ND: Normal diet, HFD: High fat diet, ATO 20: atorvastatin 20 mg/kg, F: Flaxseed 40 g/kg, FO 40: Flaxseed oil 40 mg/kg.

To analyze the histopathology of NAFLD, the H&E stained liver sections (Figure 2) were scored by a histopathologist (Table 1). The groups fed with HFD were characterized by the presence of moderate steatosis, lobular inflammation, and hepatocyte ballooning, with a moderate increase in NAFLD activity scores (Table 1). However, compared to the HFD, there was less lobular inflammation in the HFD+ATO 20, HFD+ F 40+ ATO 20, and HFD+FO 40 groups, with atorvastatin demonstrating a superior reduction compared to other treatment groups (Table 1). Compared to the HFD, the ballooning of the hepatocytes was reduced in the HFD+ATO 20 and HFD+ F 40 groups (Table 1). All intervention groups had lower NAFLD activity scores than the HFD group, which had the lowest activity score of the HFD + ATO 20 group (Table 1). 

### Most of the diet interventions decreased the levels of liver enzymes in the serum indicating reduced hepatic damage

**Figure 3:**
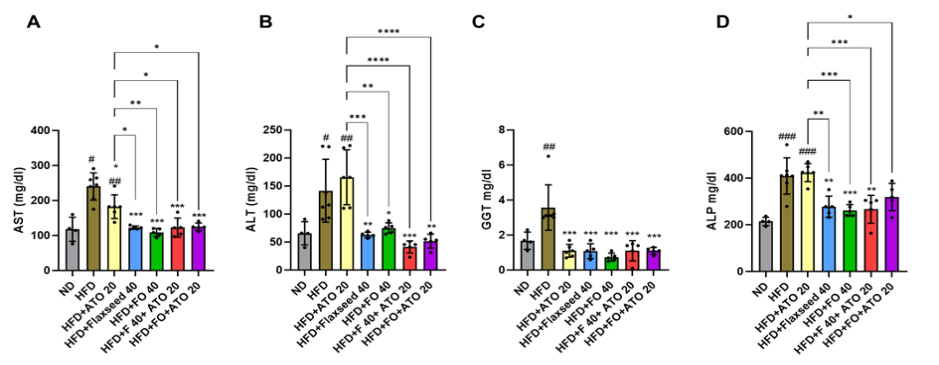
Serum levels of the liver enzymes (A: AST, B: ALT, C: GGT, D: ALP) in the study groups. ND: Normal diet, HFD: High fat diet, ATO 20: atorvastatin 20 mg/kg, F: Flaxseed 40 g/kg, FO 40: Flaxseed oil 40 mg/kg. The results are expressed as mean ± SD for each group. Individual data points within the columns are indicated with dots. ###p<0.001 compared to ND, ***p<0.001 compared to HFD. The lines connecting the columns indicate that groups differ from each other, and * denotes for p<0.05, ** for p<0.01, and *** for p<0.001. For group comparisons, we used One-Way ANOVA and Tukey post hoc test (eight rats per group).

To further characterize hepatic damage, the serum levels of liver enzymes were measured. Compared to the ND, the HFD group had higher levels of AST, ALT, GGT, and ALP (p<0.05 for all, Figure 3A-D), and the HFD+ ATO 20 group had higher AST, ALT, and ALP (p<0.01 for all, Figure 3A, B and D). Compared to the HFD, all intervention groups had lower levels of all liver enzymes (p<0.05 for all, Figure 3A-D), except the level of AST that did not differ between the HFD and HFD+ ATO 20 group (Figure 3B). To summarize, the flaxseed and combination therapies were more efficient in reducing the liver enzymes than atorvastatin, though atorvastatin monotherapy reduced more lobular inflammation and hepatocyte ballooning.

### The diet interventions improved serum lipid profile and all, except atorvastatin, decreased fasting blood glucose levels

**Figure 4:**
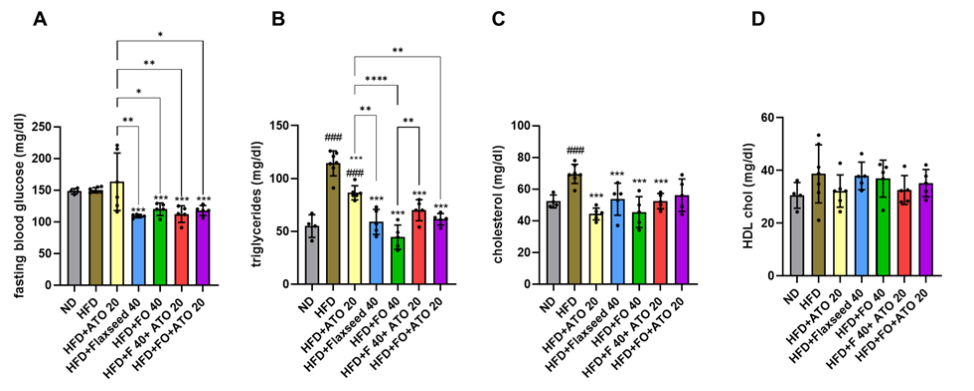
Serum levels of fasting glucose (A) and lipids (B: TG, C: Cholesterol, D: HDL-chol) in the study groups. ND: Normal diet, HFD: High fat diet, ATO 20: atorvastatin 20 mg/kg, F: Flaxseed 40 g/kg, FO 40: Flaxseed oil 40 mg/kg. The results are expressed as mean ± SD for each group. Individual data points within the columns are indicated with dots. ###p<0.001 compared to ND, ***p<0.001 compared to HFD. The lines connecting the columns indicate that groups differ from each other, and * denotes for p<0.05, ** for p<0.01, and **** for p<0.0001. For group comparisons, we used One-Way ANOVA and Tukey post hoc test (eight rats per group).

To determine the effects of the interventions on glucose and lipid metabolism, the serum levels of fasting glucose, triglycerides, total cholesterol, and HDL-chol were measured. All intervention groups except HFD+ATO 20 had lower levels of fasting blood glucose than the HFD group (p<0.001 for all, Figure 4A). Consequently, the flaxseed and combination therapies were more efficient in reducing the levels of fasting glucose than atorvastatin alone (p<0.05 for all, Figure 4A). Compared to the ND, the HFD and HFD+ATO 20 groups had higher levels of triglycerides (p<0.001 for both, Figure 4B), while all interventions decreased the levels compared to the HFD (p<0.001 for all, Figure 4B). The flaxseed and combination therapies were more efficient in reducing the levels of triglycerides than atorvastatin alone (p<0.01 for all, Figure 4B). All diet interventions, except HFD+ FO+ATO 20 reduced the levels of total cholesterol, when compared to the HFD group (p<0.001 for all, Figure 4C). The groups did not differ from each other in the levels of HDL-chol (Figure 4D).

## Discussion

In the current study, we investigated whether dyslipidemia and liver injuries associated with NAFLD could be modulated by atorvastatin and flaxseed (whole seeds or oil). The results showed that atorvastatin, flaxseed, or a combination therapy can be effective in reducing the amount of mesenteric fat and fat around the liver as well as NAFLD activity scores. This is in agreement with previous studies (Cioboată et al. 2017; Doumas et al. 2018; Yu et al. 2018). However, the combination therapies were more effective in improving NAFLD-associated serum lipid profile and liver injury markers than atorvastatin monotherapy. 

While all interventions reduced the amount of fat around the liver and the NAFLD activity scores, atorvastatin monotherapy was most efficient in reducing lobular inflammation and hepatocyte ballooning. Although statins are primarily used to lower cholesterol, it is known that they have beneficial effects on hepatic inflammation and fibrosis, which is in line with our results (Schierwagen et al. 2017). Interestingly, the effects of flaxseed on NAFLD have recently been evaluated in a clinical trial (Yari et al. 2021). Like our study in rats, the trial showed that flaxseed supplementation for 12 weeks could improve lipid and glucose metabolism, and reduce inflammation and hepatic steatosis. As a result, and most importantly, our findings from a preclinical rat model provide more foundations for future clinical trials attempting to treat NAFLD with flaxseed.

We show that all flaxseed and flaxseed oil-based interventions reduced the levels of serum triglycerides and total cholesterol. While the cholesterol-lowering effects of atorvastatin are very well known, previous studies have demonstrated a hypocholesterolemic and anti-atherogenic role for flaxseed (Kajla et al. 2015; Pan et al. 2009). According to Bhathena and co-workers, in both lean and obese rats, flaxseed supplementation significantly reduced triglyceride levels and fat deposition in the liver (Bhathena et al. 2003). Studies are showing that flaxseed can decrease fatty acid generation by inhibiting lipogenic enzymes, like lipogenic transcriptional factor SREBP1 (sterol regulatory element-binding protein) and FAS (fatty acid synthase) (Devarshi et al. 2013). PPARα (Peroxisome proliferator-activated receptor alpha) regulates fatty acid metabolism, and ALA (α-Linolenic acid) is a natural PPARα ligand, binding to and activating PPARα to elevate gene expression and enzyme activity associated with hepatic fatty acid oxidation (Devarshi et al. 2013; Murase et al. 2005). In the future, the mechanisms through which flaxseed and the combination therapies used in our study improve hepatic health, should be studied more.

Visceral fat is significantly more likely than subcutaneous fat to develop cardiovascular and metabolic problems, which are mediated by inflammatory adipokines (Marchesini et al. 2019). The amount of mesenteric fat is shown to be an independent determinant of all components of metabolic syndrome (Liu et al. 2006b) and a risk factor for fatty liver (Liu et al. 2006a). Therefore, it was an important finding that all interventions in this study were able to reduce the amount of mesenteric fat compared to the HFD. Contrary to our current results, it has been reported that atorvastatin increases the size of visceral adipocytes without affecting the total body fat mass (Mondragón-García et al. 2019). The mesenteric fat is anatomically connected to the gut and is important in mediating inflammation (Peyrin-Biroulet et al. 2012). As it increases intestinal integrity, it has been shown to be protective of NAFLD, which is contrary to our results (Wu et al. 2018). 

Regarding glycemic control, whole flaxseed was able to reduce the levels of fasting blood glucose when compared to the HFD group, making the monotherapy more efficient than the other treatments. The hypoglycemic potential of flaxseed may be due to its high fiber content. This can be due to the high soluble fiber content of the seeds, particularly in the mucilage gums, which slows food digestion and reduces the absorption of some nutrients such as sugar (Goyal et al. 2014; Soliman 2019). An observational study demonstrated that the daily consumption of 10 grams of flaxseed powder by type 2 diabetic individuals decreased fasting blood glucose by 19.7% compared with the control group. Mani and colleagues also observed a favorable decrease in total cholesterol and triglycerides (Mani et al. 2011). Similarly, in type 2 diabetes patients, Thakur et al. showed a reduction by 12% of fasting blood glucose after daily consumption of 5 grams of flaxseed compared with a control group (Thakur et al. 2009). In individuals with prediabetes, similar results were observed. The daily consumption of flaxseed (13 grams) decreased serum levels of glucose and insulin and increased insulin sensitivity in obese or overweight individuals with pre-diabetes (Hutchins et al. 2013). Though according to these studies, flaxseeds appear to be beneficial for glycemic control, some studies indicate the opposite (Barre et al. 2008; Mohammadi-Sartang et al. 2018).

Regarding the serum markers of liver injury, a significant increase was detected in the levels of ALT, AST, ALP, and GGT in rats fed with HFD when compared to the ND, which is in agreement with previous clinical and pre-clinical studies (Hadizadeh et al. 2017; Pereira et al. 2021; Silvares et al. 2016). While atorvastatin only reduced GGT levels, the ingestion of flaxseed or flaxseed oil alone or in combination therapies reduced the levels of all liver enzymes. In agreement, a study by Zhang et al. demonstrated that flaxseed oil supplementation caused to significant reduction in plasma ALT and AST and alleviation liver damage caused by chronic ethanol feeding (Zhang et al. 2017). When compared to untreated hypertensive-fed rats, flaxseed treatment caused a significant decrease in serum ALT and AST levels in hypertensive Wistar rats (Al-Bishri 2013). In addition, flaxseed oil treatment in hypercholesterolemic rats is shown to lower serum levels of liver enzymes and improve the antioxidant enzymatic status of liver tissue (Hussein et al. 2016). 

Taken together, these studies and the current study show an important hepatoprotective effect of flaxseed.

 In conclusion, compared to the HFD all interventions reduced mesenteric fat mass, and all, except HFD+FO 40 +ATO 20, reduced the amount of fat around the liver. Flaxseed therapies alone or in combination with atorvastatin efficiently improved NAFLD-associated liver injuries and dyslipidemia, while the main effect of atorvastatin was to reduce lobular inflammation and hepatocyte ballooning.

**Table 1:** Histological Scoring of NAFLD in the study group, and the components of NAFLD activity score (NAS)

NAS activity component	ND	HFD	HFD+ ATO 20	HFD+ Flaxseed 40	HFD+ FO 40	HFD+ F 40 +ATO 20	HFD+ FO 40 +ATO 20
**Steatosis**	0	2	1	1	1	1	1
**Lobular Inflammation**	0	2	0	2	1	1	2
**Hepatocyte Ballooning**	0	1	0	0	1	1	1
**SCORE**	0	5	1	3	3	3	4
